# Evaluation of tetrachloroethylene (PCE) and its degradation products in human exhaled breath and indoor air in a community setting

**DOI:** 10.1088/1752-7163/ad67fd

**Published:** 2024-08-08

**Authors:** Jung Hyun Lee, Alaina K Bryant, Marwan Alajlouni, Brandon E Boor, Antonios Tasoglou, Sa Liu

**Affiliations:** 1 School of Health Sciences, Purdue University, West Lafayette, IN, United States of America; 2 Lyles School of Civil Engineering, Purdue University, West Lafayette, IN, United States of America; 3 RJ Lee Group Inc., Monroeville, PA, United States of America; 4 These authors contributed equally to this work.

**Keywords:** tetrachloroethylene, exhaled breath, Superfund site, community setting, PTR-TOF-MS

## Abstract

Tetrachloroethylene (PCE) is a widely utilized volatile chemical in industrial applications, including dry cleaning and metal degreasing. Exposure to PCE potentially presents a significant health risk to workers as well as communities near contamination sites. Adverse health effects arise not only from PCE, but also from PCE degradation products, such as trichloroethylene (TCE) and vinyl chloride (VC). PCE, TCE, and VC can contaminate water, soil, and air, leading to exposure through multiple pathways, including inhalation, ingestion, and dermal contact. This study focused on a community setting in Martinsville, Indiana, a working-class Midwestern community in the United States, where extensive PCE contamination has occurred due to multiple contamination sites (referring to ‘plumes’), including a Superfund site. Utilizing proton transfer reaction time-of-flight mass spectrometry (PTR-TOF-MS), PCE, TCE, and VC concentrations were measured in the exhaled breath of 73 residents from both within and outside the plume areas. PCE was detected in 66 samples, TCE in 26 samples, and VC in 68 samples. Our results revealed a significant positive correlation between the concentrations of these compounds in exhaled breath and indoor air (Pearson correlation coefficients: PCE = 0.75, TCE = 0.71, and VC = 0.89). This study confirms the presence of PCE and its degradation products in exhaled breath in a community exposure investigation, demonstrating the potential of using exhaled breath analysis in monitoring exposure to environmental contaminants. This study showed the feasibility of utilizing PTR-TOF-MS in community investigations to assess exposure to PCE and its degradation products by measuring these compounds in exhaled breath and indoor air.

## 1. Introduction

Tetrachloroethylene (PCE) (CAS Registry Number 127-18-4; C_2_Cl_4_; molecular weight 165.83 g mol^−1^) is a chlorinated volatile organic compound (VOC) used as an industrial solvent in dry cleaning and metal degreasing. Exposure to PCE can occur in workplaces and communities near PCE contamination sites [1, 2]. PCE exposure is associated with various adverse health effects in humans, including neurobehavioral effects [3], renal dysfunction [4, 5], hepatotoxicity [6, 7], immunotoxicity [8], reproductive problems [9, 10], and maternal toxicity [11]. Notably, PCE is classified as probably carcinogenic to humans (Group 2A) by the International Agency for Research on Cancer (IARC) [12]. Furthermore, PCE’s degradation products, including trichloroethylene (TCE) and vinyl chloride (VC), are classified as IARC Group 1A carcinogens [13, 14].

The United States Environmental Protection Agency (U.S. EPA) has found PCE at 949 out of 1,854 Superfund sites [15]. PCE is measured in groundwater, soil vapor, indoor air, and sub-slab air to evaluate environmental contamination, but these measurements may not fully represent human exposure due to complex environmental exposure pathways and variations in physical activities [16]. Inhalation has been identified as one of the most common routes of PCE exposure. Since PCE can readily evaporate from groundwater and soil, infiltrate buildings, and contaminate the indoor atmospheric environment, people can be easily exposed from their homes or workplaces through inhalation [17, 18]. Ingestion of PCE-contaminated water and dermal contact are also potential threats to public health. The multifaceted nature of PCE exposure underscores the need to employ biomonitoring techniques to evaluate human exposure that could happen through multiple pathways in environmental settings.

Biomonitoring, particularly exhaled breath analysis, is effective for evaluating PCE exposure [17]. Exhaled breath sampling presents a non-invasive and convenient method for sample collection [19–21]. Exhaled breath analysis is based on the equilibrium between alveolar air and pulmonary capillary blood, reflecting PCE levels in the bloodstream [22, 23]. PCE can enter the bloodstream through ingestion, dermal contact, or inhalation. In particular, 80%–100% of PCE intake is unmetabolized and eliminated as exhaled breath within days [24–27]. Exhaled breath analysis reliably indicates PCE exposure, correlating with detected PCE levels in the residential indoor air of people living near dry cleaners [28–35].

Sample collection and analysis are key to quantifying PCE in exhaled breath. Conventional methods using sample bags or canisters and gas chromatography-mass spectrometry (GC-MS) are limited by potential PCE degradation or contamination during transportation [17, 27, 36, 37]. To prevent this, in-situ mobile techniques such as photoionization detectors, electrochemical sensors, metal oxide sensors, electronic noses, UV spectroscopy, chemiluminescence, miniaturized gas chromatography, and portable mass spectrometry have been developed [38–45]. Nevertheless, the detection limits of these methods may prove insufficient for quantifying PCE concentrations in community settings [39–42]. To address these challenges, an emerging instrument can be used, which is the proton transfer reaction time-of-flight mass spectrometer (PTR-TOF-MS), a highly sensitive, portable, real-time monitoring device [46–54]. PTR-TOF-MS can measure hundreds to thousands of VOCs, including PCE, with a low detection limit (parts per trillion (ppt) level) in real-time (1 Hz). Its size allows for transportation in a vehicle, enabling on-site exhaled breath analysis in community settings. The PTR-TOF-MS, commonly used in various indoor air and exposure studies [46–54], has not yet been employed to measure PCE and its degradation products via exhaled breath analysis in community settings. This study aims to assess PCE exposure in residents of contaminated areas using PTR-TOF-MS, focusing on both PCE and its degradation products, TCE and VC.

The field measurement campaign took place in Martinsville, Indiana, a city with four known PCE plumes, one designated as a Superfund site (hereinafter referred to as ‘Superfund Site’). Initial investigations conducted by the Indiana Department of Environmental Management (IDEM) showed elevated PCE concentrations in groundwater and soil, which have since decreased in some areas due to remediation efforts [55]. The study targeted children aged 6 to 11, given their possible vulnerability to PCE exposure [56, 57], as part of a pilot epidemiological study investigating the impact of PCE exposure on children’s neurobehavioral performance. Community members were trained to assist with breath collection and communicate study objectives to participants, leveraging their local knowledge to encourage participation.

**Figure 1.**
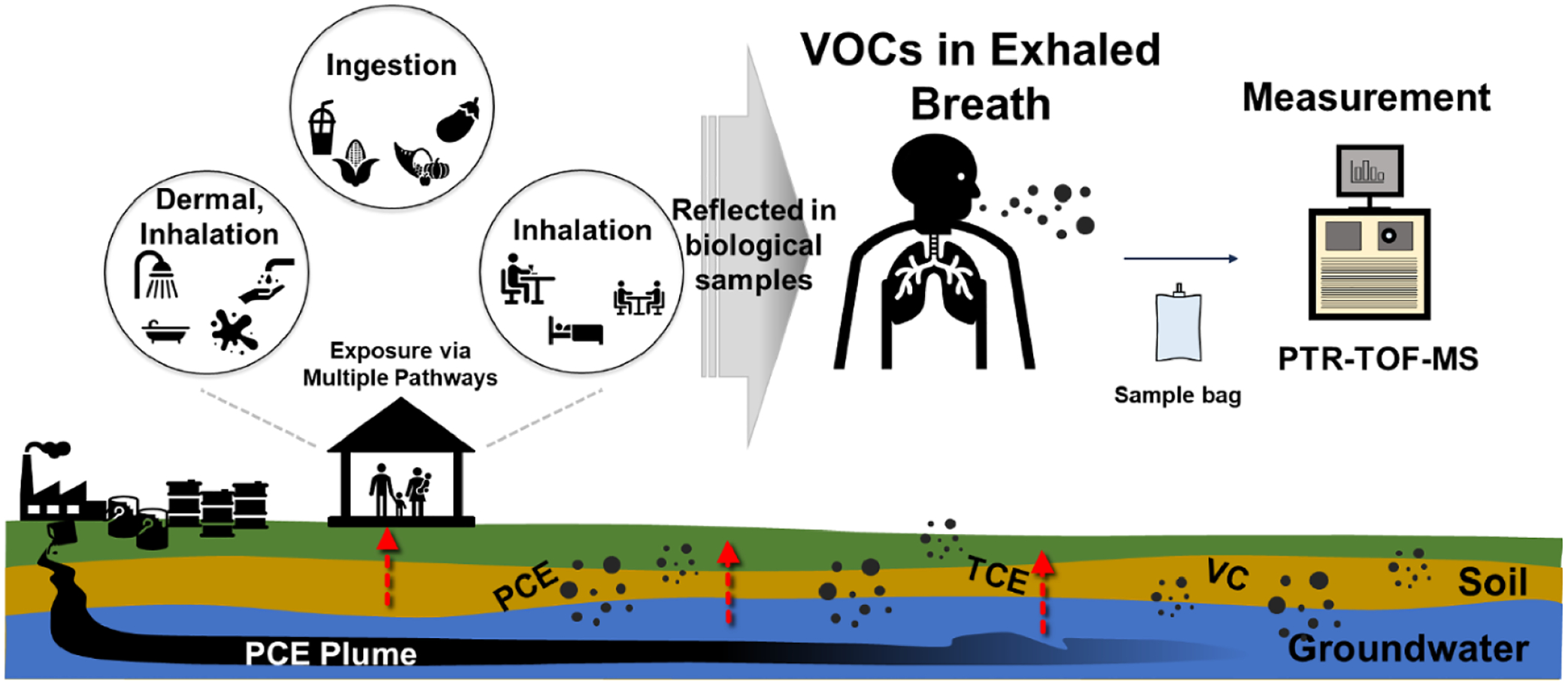
Exposure to PCE via multiple pathways and real-time VOC measurement in exhaled breath via PTR-TOF-MS analysis.

This paper reports the analysis of exhaled breath and indoor air using PTR-TOF-MS to understand PCE exposure within and outside contaminated areas (figure 1). This project also examined how the concentrations of PCE and its degradation products in exhaled breath varied during the day (from 10:00 A.M. to 08:00 P.M.) potentially due to activity patterns, fluctuations in environmental concentrations, and physiological rhythms [56]. A total of 105 samples of exhaled breath from 73 participants and 13 indoor air samples were collected in residences both within and outside the contaminated areas, thereby evaluating correlations between PCE concentrations in exhaled breath and indoor air, as well as that for PCE degradation products (TCE and VC).

## 2. Materials and methods

### 2.1. Study location, participants, and sample collection

This study was conducted in Martinsville, Indiana, which has four groundwater contamination sites, or ‘plumes’. One plume originated from a dry-cleaning facility operating from 1986 to 1991, leading to PCE contamination in groundwater, soil, and air. PCE can infiltrate buildings through foundation cracks, increasing indoor air concentrations [22] and exposing residents to health risks such as neurological impairment, liver and kidney damage, and cancer. The contamination covers over 60 acres (around 0.24 km^2^) and includes significant contaminants like PCE, TCE, VC, methyl ethyl ketone, acetone, and 1,4-dioxane [17]. Three additional known groundwater contamination sites, namely, the O’Neal, Twigg, and Harman-Becker plumes, are currently undergoing investigation and remediation processes conducted by IDEM. Participant recruitment was supported by Martinsville community members and the community advisory board (CAB), who regularly met to discuss exposure concerns and to facilitate the study [17]. The pilot epidemiological study, approved by the Purdue University Institutional Review Board (IRB-2021–1507), recruited 73 participants for whom exposure assessment was conducted.

Participants received instructions a week before sampling to avoid factors affecting breath composition, such as dairy consumption [58], pesticides, herbicides, nail polish, and swimming pools. Sample collection took place on weekend mornings (08:00 A.M. to 12:00 P.M.) using sample bags (1-liter polypropylene fitted breath-gas analysis bags, Tedlar^®^ material, SKC Inc., Cat. No. 249–01, USA). The sample bag was connected to a single-use plastic mouthpiece (SKC Inc., Cat. No. P20054, USA) [59] to prepare the sampling of exhaled breath. Trained CAB members assisted in exhaled breath collection at participant’s homes. Participants completed a demographic questionnaire and practiced the breathing technique before sampling by watching a video made by the research team. Participants exhaled into the sample bags following specific instructions to reach 80%–90% of the bag capacity. Samples were immediately put into a cooler to prevent chemical degradation and potential contamination. Subsequently, exhaled breath samples were transported to a mobile laboratory positioned nearby in the community for the on-site analysis using a commercial PTR-TOF-MS instrument (PTR-TOF 4000, Ionicon Analytik Ges.m.b.H., Innsbruck, Austria). In addition, repeated samples were collected from six participants throughout the day (from 10:00 A.M. to 08:00 P.M.) with 3–4 measurements per participant (two participants were sampled 3 times, and four participants were sampled 4 times) to evaluate diurnal variations in VOC concentrations in exhaled breath.

Indoor air sampling was conducted in 11 homes of 13 participants, including siblings, to evaluate the correlation between concentrations of PCE and its degradation products in indoor air and exhaled breath using the PTR-TOF-MS. The mobile laboratory, equipped with a sampling manifold, measured VOC concentrations in real-time, with bedrooms chosen for indoor air sampling as they are where participants spend most of their time [60]. During the indoor air testing, a sampling line made of perfluoroalkoxy tubing with an outer diameter of 0.9525 cm was used. To remove airborne particles, a polytetrafluoroethylene filter (1 *μ*m pore size) was installed at the inlet of the sampling line.

### 2.2. PTR-TOF-MS

The PTR-TOF-MS enables real-time measurement of VOCs in exhaled breath samples, utilizing soft chemical ionization to measure individual VOCs at ppt levels, and features fast response times for online analysis of end-tidal exhaled breath in field assessments [61]. VOCs in exhaled breath can react with reagent ions, such as hydronium $\left({\text{H}}_{3}{\text{O}}^{+}\right)$ and oxygen $\left({\text{O}}_{2}^{+}\right)$, in the drift tube section of the PTR-TOF-MS. When using ${\text{O}}_{2}^{+}$, as done in this study to better detect chlorinated VOCs such as PCE, this leads to a soft chemical ionization of the VOCs through a charge-transfer reaction with ${\text{O}}_{2}^{+}$, as shown in equation (1) [62]

(1) $$O_{2}^{+}+R\to R^{+}+O_{2}.$$

The PTR-TOF-MS detected ionized VOCs $\left({\text{R}}^{+}\right)$ in the mass range from m/z 20–450 with a mass resolution exceeding 4000 m/Δm. Operational parameters of the PTR-TOF-MS were informed by our previous indoor air and exposure measurements with the PTR-TOF-MS [52, 53] and set as follows: an inlet flow rate of 700 ml min^−1^, an electric field strength to gas number density ratio (E/N) of 139 Td, an inlet temperature of 80 °C, a reaction chamber pressure of 2.2 mbar, a reaction chamber temperature of 70 °C, and a reaction chamber voltage of 600 V. Daily calibrations of the PTR-TOF-MS were performed using known standard gas mixtures (Apel-Riemer Environmental Inc., USA) containing 19 different compounds commonly measured in indoor air, including PCE, TCE, VC, toluene, 1,2-dichloroethylene (1,2 DCE), acetone, benzene, isoprene, and methyl ethyl ketone. In the calibration process, the standard gas mixtures were diluted with VOC-free air (Matheson Tri-Gas Inc., USA) using a mass flow controller, achieving concentrations ranging from 2 to 50 parts per billion (ppb). The mass-dependent ion transmission of the PTR-TOF-MS was assessed based on the information derived from the daily calibrations. Furthermore, calibration curves were generated for each VOC to convert the instrument signal in counts per second (cps) to concentration (ppb).

Exhaled breath samples were connected to the instrument for a duration of 100 s, during which mass spectra were recorded at a frequency of 1 Hz. This approach facilitated the observation of spectrum stability over consecutive results within the 100 s sampling segment. The obtained mass spectra underwent analysis using the instrument’s proprietary software (PTR-MS Viewer, Version 3.2.2, Ionicon Analytik Ges.m.b.H., Innsbruck, Austria). Indoor air was sampled and analyzed simultaneously. The sampling duration for indoor air was at least 10 min (600 s).

### 2.3. PTR-TOF-MS data acquisition

Post sample data processing involves mass scale calibration, peak identification, noise reduction, calculation of concentrations (in ppb), and time-series analysis. Mass scale calibration and peak identification were performed using raw data and PTR-MS Viewer. To ensure accuracy, the instrument executed a mass scale calibration process by matching the m/z signals of the specific compounds identified. The m/z values of 165.87 (PCE), 129.91 (TCE), and 61.99 (VC) were used to identify PCE, TCE, and VC, respectively [63]. Several compounds, including PCE, were calibrated using the standard gas mixture with known concentrations. The concentrations of these compounds were calculated using the measured sensitivity (ncps/ppb) of each VOC. The measured sensitivity was obtained from calibration curves as shown by equation (2) [64, 65]

(2) $$\text{Sensitivity}(\text{ncps}/ppb)=\frac{{ion}_{R^{+},\text{cal}}(cps)-{\text{Background}}_{\text{cal}}(cps)}{{ion}_{{O_{2}}^{+},\text{cal}}(cps)}\cdot \frac{1}{{\text{Concentration}}_{\text{cal}}(ppb)}10^{6}$$

where ${ion}_{R^{+},\text{cal}}$ is the measured cps for the diluted standard gas mixture, ${ion}_{{O_{2}}^{+},\text{cal}}$ is the measured cps for the reagent ion ${\text{O}}_{2}^{+},{Background}_{\text{cal}}$ is the measured cps of VOC-free air, and ${Concentration}_{cal}$ is the known ppb of the diluted standard gas mixture. The calculation of concentrations was applied to the time-series data of the measurement, resulting in time-series concentrations of VOCs. To specify the VOCs from exhaled breath, the acetone signal was used as an indicator of exhaled breath [61]. Therefore, the time points of exhaled breath into the PTR-TOF-MS can be specified by observing the acetone time-series. Specific time points of VOC concentrations within the total time-series data were extracted and used for data analysis. The indoor air data was analyzed based on the measurement time, because indoor air measurements were performed in real-time. The average value was calculated by selecting a 20 s interval from the end of the sampling period.

### 2.4. Data analysis

In this study, statistical methods were employed to explore correlations between concentrations of PCE and its degradation products in exhaled breath and indoor air to evaluate the relationship between these two key exposure metrics. The data analysis primarily involved regression analysis and Pearson correlation testing. Regression analysis was utilized to assess the relationship between concentrations of PCE and its degradation products in exhaled breath and indoor air. Calculations were performed using Microsoft Excel (Excel 365, Microsoft Corporation, Redmond, WA, USA).

## 3. Results

### 3.1. Comparison of PCE, TCE, and VC concentrations in exhaled breath of participants located inside and outside of the plume

**Figure 2.**
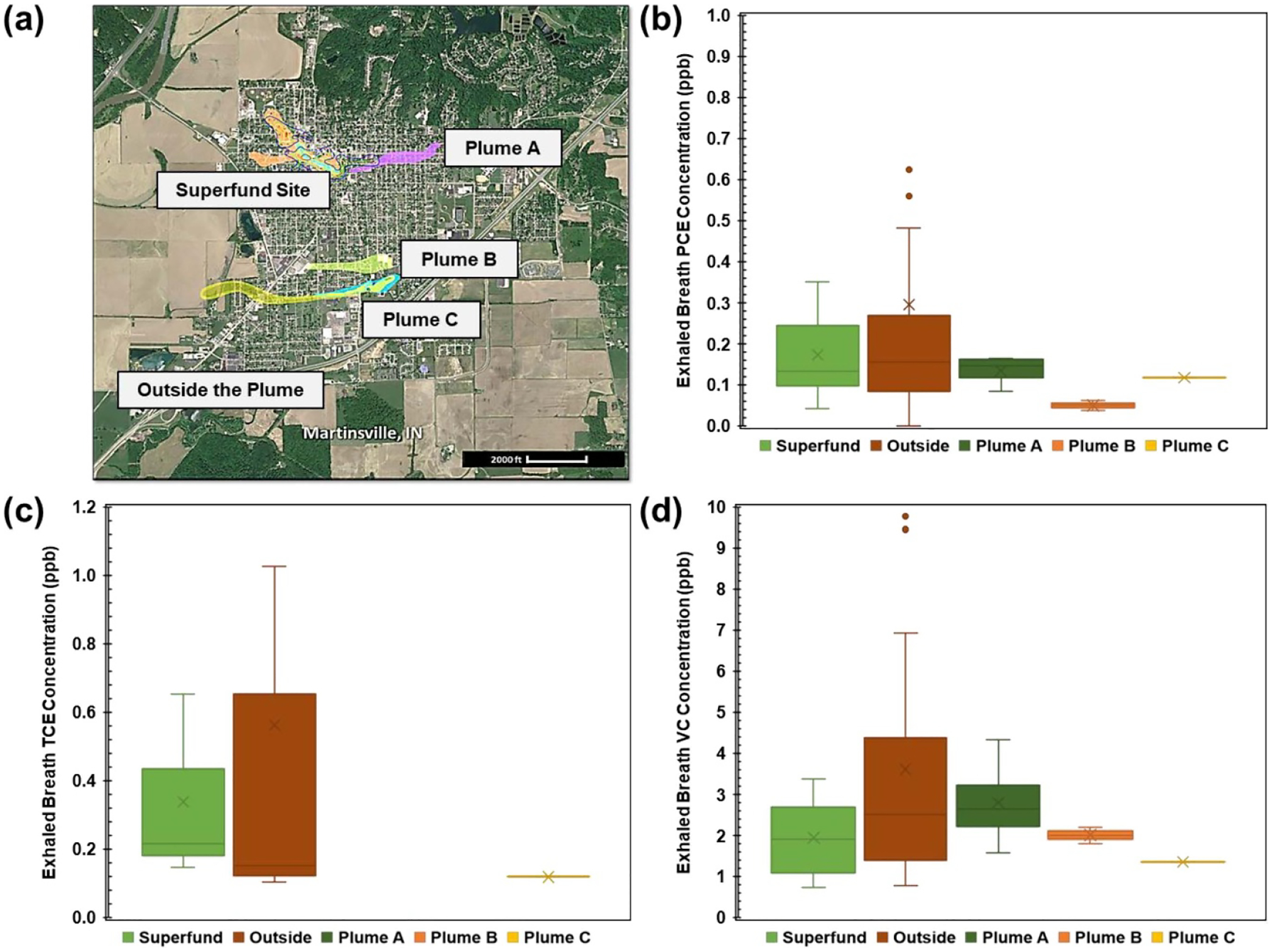
Sampling site and exhaled breath PCE, TCE, and VC concentrations (a) sampling site locations for the Superfund Site, Plume A, Plume B, Plume C, and Outside the Plume (Google map modified) (b) Exhaled breath PCE concentration from the Superfund Site (N=7, mean = 0.17 ppb, median = 0.13 ppb, 25% = 0.10 ppb, 75% = 0.24 ppb), Plume A (N=4, mean = 0.13 ppb, median = 0.15 ppb, 25% = 0.12 ppb, 75% = 0.16 ppb), Plume B (N=2, mean = 0.05 ppb, median = 0.05 ppb, 25% = 0.04 ppb, 75% = 0.06 ppb), Plume C (N=1, mean = 0.12 ppb, median = 0.12 ppb, 25% = 0.12 ppb, 75% = 0.12 ppb), and Outside the Plume (N=52, mean = 0.30 ppb, median = 0.16 ppb, 25% = 0.08 ppb, 75% = 0.27 ppb) (c) Exhaled breath TCE concentration from the Superfund Site (N=3, mean = 0.34 ppb, median = 0.22 ppb, 25% = 0.18 ppb, 75% = 0.43 ppb), Plume A (no samples), Plume B (no samples), Plume C (N=1, mean = 0.12 ppb, median = 0.12 ppb, 25% = 0.12 ppb, 75% = 0.12 ppb), and Outside the Plume (N=22, mean = 0.56 ppb, median = 0.15 ppb, 25% = 0.12 ppb, 75% = 0.65 ppb) (d) Exhaled breath VC concentration from the Superfund Site (N=7, mean = 1.94 ppb, median = 1.91 ppb, 25% = 1.09 ppb, 75% = 2.69 ppb), Plume A (N=4, mean = 2.80 ppb, median = 2.65 ppb, 25% = 2.23 ppb, 75% = 3.22 ppb), Plume B (N=2, mean = 2.01 ppb, median = 2.01 ppb, 25% = 1.91 ppb, 75% = 2.11 ppb), Plume C (N=1, mean = 1.36 ppb, median = 1.36 ppb, 25% = 1.36 ppb, 75% = 1.36 ppb), and Outside the Plume (N=54, mean = 3.61 ppb, median = 2.52 ppb, 25% = 1.39 ppb, 75% = 4.37 ppb). *N* is the number of samples that have a detectable concentration.

A total of 73 participants residing in various locations, including the Superfund Site (n=7), Plume A (n=4), Plume B (n=2), Plume C (n=1), and Outside the Plume (n=59), were included in the study. In figure 2(a), the Superfund Site, Plume A, Plume B, Plume C, and Outside the Plume are marked on a map of Martinsville. The average concentrations of PCE, TCE, and VC in exhaled breath are shown in the caption of figure 2. PCE was detected in a total of 66 exhaled breath samples, TCE was detected in 26 exhaled breath samples, and VC was detected in 68 exhaled breath samples.

Comparing the four plume regions (excluding Outside the Plume), residents in the Superfund Site exhibited the highest average PCE concentration in exhaled breath (figure 2(b)). However, the results from Outside the Plume suggest that PCE exposure may occur through multiple pathways, indicating that participants may be influenced not only by their residential locations, but also by the spaces they occupy during the daytime. In addition, PCE can be used in a variety of ways, including in paint, furniture, and cleaning products, thus there may be other PCE sources in the residents’ homes that may contribute to the observed exposures (see the following discussion section). Furthermore, given that most of the participants were primary school students and considering the 2–3-day half-life of PCE in the body, it is imperative to consider where they spend the majority of their time during the week [22]. In figure 2(c), TCE was not detected from Plume A and Plume B. The results from the Outside the Plume area showed the highest average concentration (mean = 0.56 ppb). The median value (0.15 ppb) was lower than the Superfund Site median value (0.22 ppb), so half of the samples had values less than 0.15 ppb. In figure 2(d), the results for VC from Outside the Plume had the highest average concentration. Comparing plume regions, residents in Plume A showed the highest average VC concentration in exhaled breath. Collectively, these results demonstrate that the majority of residents throughout Martinsville are exposed to PCE and its degradation products, likely due to a combination of plume- and non-plume sources near their homes and other locations they occupy during the daytime. Mobile exhaled breath analysis of chlorinated VOCs with the PTR-TOF-MS provided a useful basis for tracking these exposures and their spatial variations across Martinsville.

### 3.2. Changes in PCE, TCE, and VC concentrations in exhaled breath during the daytime

**Figure 3.**
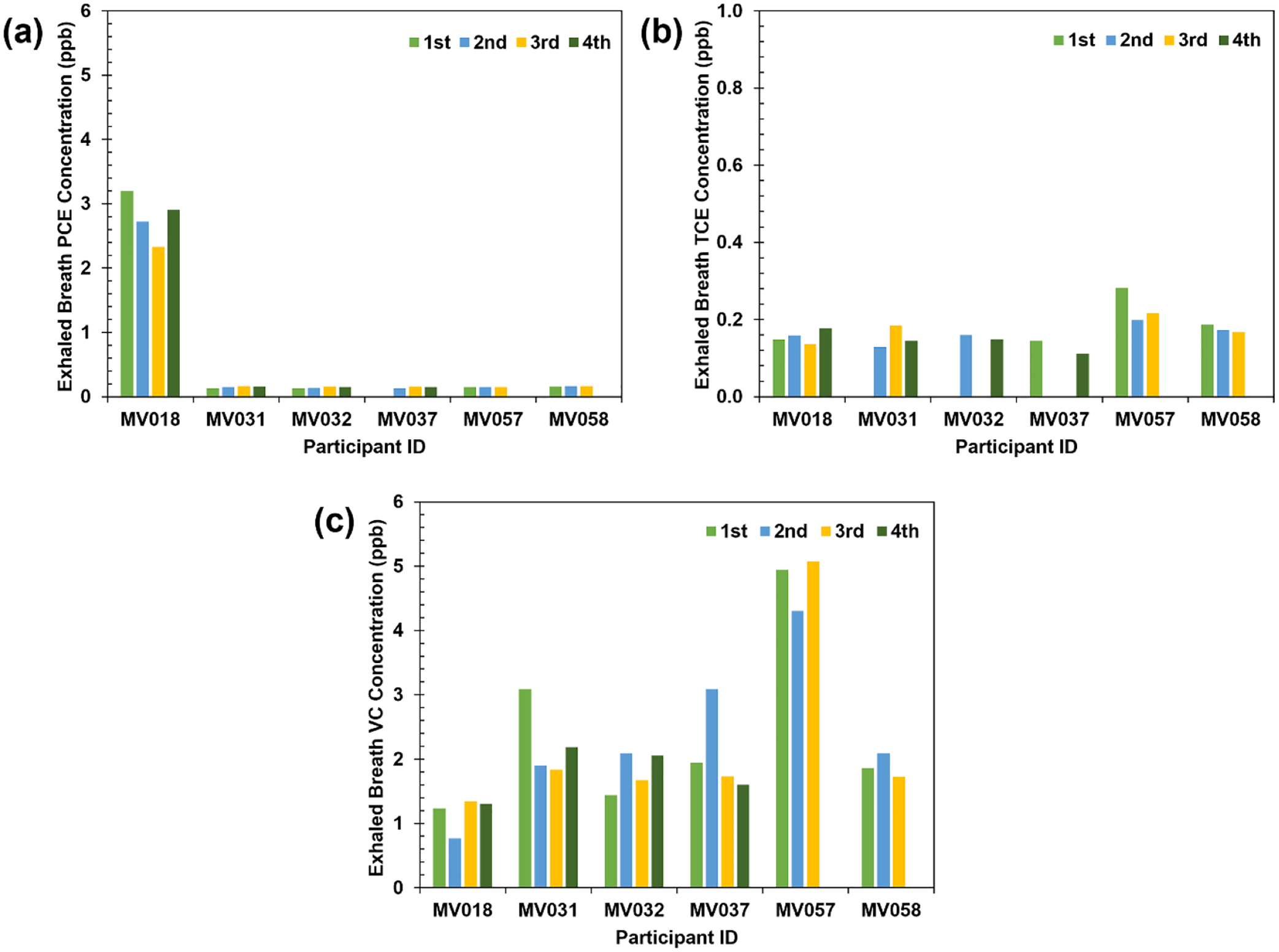
Changes in PCE, TCE, and VC concentrations in exhaled breath during the daytime for six participants.

To assess the diurnal variation in PCE, TCE, and VC concentrations in exhaled breath, six participants who lived Outside the Plume were monitored from 10:00 A.M. to 8:00 P.M. (figure 3). Their participation was voluntary. Two participants were sampled 3 times, and four participants were sampled 4 times. Their IDs are noted as MV0## (table 1). As illustrated in figure 3, concentrations of PCE, TCE, and VC in exhaled breath were generally consistent throughout the daytime, with periodic elevations observed for a few samples, most notably for VC (MV031, MV037, and MV057). Average PCE concentrations for MV018, MV031, MV032, MV037, MV057, and MV058 were 2.79 ppb, 0.15 ppb, 0.14 ppb, 0.14 ppb, 0.15 ppb, and 0.16 ppb, respectively (table 1). There was no significant difference (p>0.05) between the sampling orders. Average TCE concentrations for MV018, MV031, MV032, MV037, MV057, and MV058 were 0.15 ppb, 0.15 ppb, 0.15 ppb, 0.13 ppb, 0.23 ppb, and 0.18 ppb, respectively (table 1). There was no significant difference (p>0.05) between the sampling orders. Average VC concentrations for MV018, MV031, MV032, MV037, MV057, and MV058 were 1.16 ppb, 2.25 ppb, 1.81 ppb, 2.09 ppb, 4.78 ppb, and 1.89 ppb, respectively (table 1). There was no significant difference (p>0.05) between the sampling orders. Notably, MV031 exhibited similar concentrations to MV032 (siblings residing in the same house). Also, MV057 exhibited similar concentrations to MV058 (siblings residing in the same house). The correlation coefficient between the average concentrations of PCE and TCE was −0.155, between PCE and VC was −0.456, and between TCE and VC was 0.838.

### 3.3. Comparison of PCE, TCE, and VC concentrations in exhaled breath and indoor air

**Figure 4.**
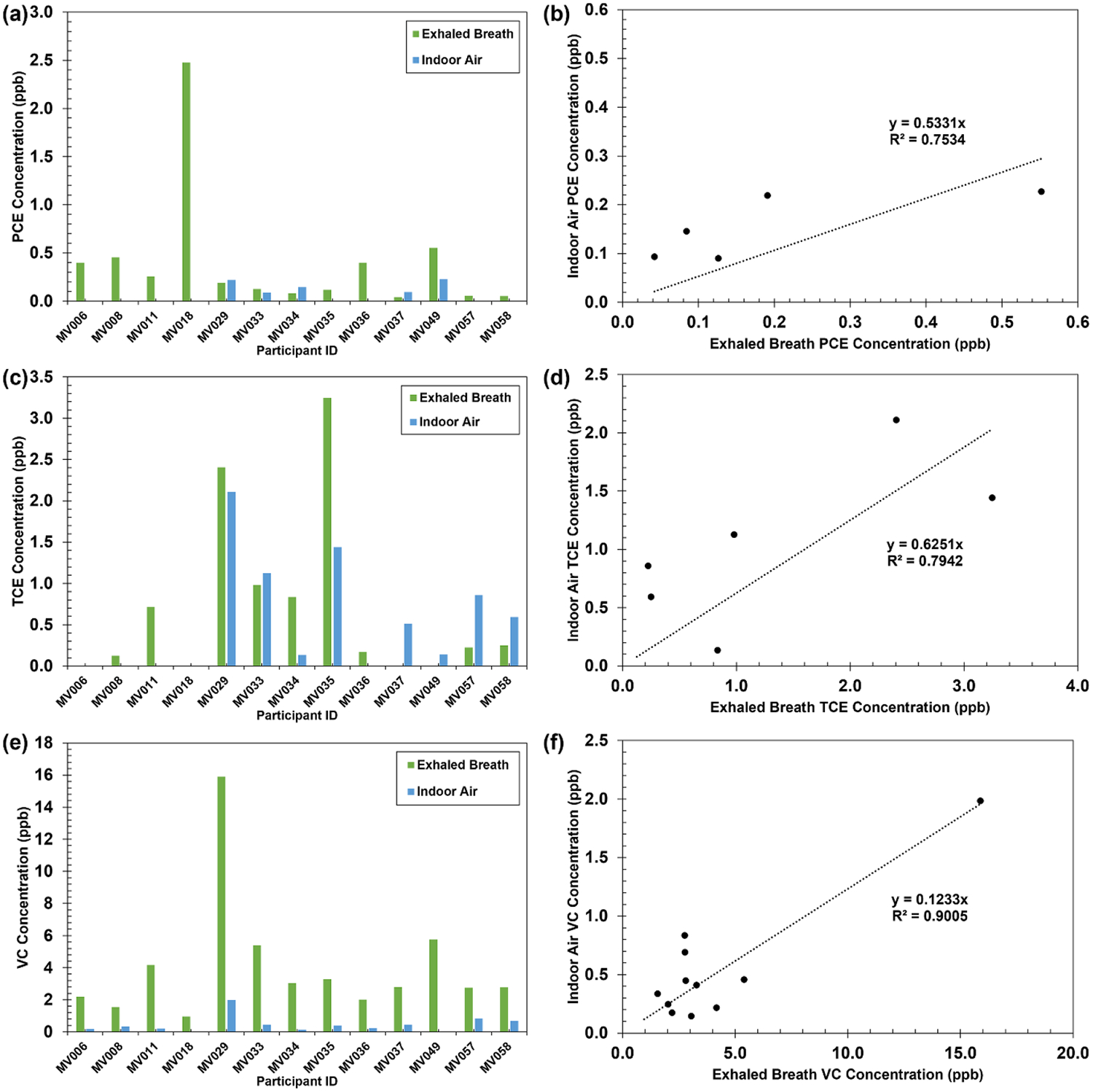
Comparison of PCE, TCE, and VC concentrations in exhaled breath and indoor air: (a) PCE concentrations in exhaled breath and indoor air (the PCE concentration in indoor air of MV008 was considered an outlier and was removed), (b) PCE linear regression (the PCE concentration in indoor air of MV008 was considered an outlier and was removed), (c) TCE concentration in exhaled breath and indoor air, (d) TCE linear regression, (e) VC concentration in exhaled breath and indoor air, and (f) VC linear regression.

To examine possible correlations between PCE, TCE, and VC concentrations in indoor air and exhaled breath, indoor air sampling was conducted at 11 homes of 13 participants, including siblings (figure 4). Indoor air was measured in the participants’ bedrooms. During the measurements, VOC signals were monitored in real-time by the PTR-TOF-MS. Once a stable signal was confirmed, sampling was performed for more than 2 min, and the average value was taken. PCE was detectable in indoor air in 6 out of 13 bedrooms, with concentrations ranging from 0.09 ppb to 0.23 ppb, except for MV008 (2.37 ppb), while exhaled breath concentrations ranged from 0.04 ppb to 2.48 ppb (figure 4(a)). Results indicate a positive correlation between exhaled breath and indoor air PCE concentrations (Pearson correlation coefficient = 0.7479). Linear regression analysis yielded a relationship between exhaled breath and indoor air concentrations (figure 4(b), $y=0.5331x,R^{2}=0.7534$). TCE was detectable in indoor air in 7 out of 13 bedrooms, with concentrations ranging from 0.14 ppb to 2.11 ppb, while exhaled breath concentrations ranged from 0.12 ppb to 3.25 ppb (figure 4(c)). Results indicate a positive correlation between exhaled breath and indoor air TCE concentrations (Pearson correlation coefficient = 0.7134). Linear regression analysis yielded a relationship between exhaled breath and indoor air concentrations (figure 4(d), $y=0.6251x,R^{2}=0.7942$). VC was detectable in indoor air in 11 out of 13 bedrooms, with concentrations ranging from 0.14 ppb to 1.99 ppb, while exhaled breath concentrations ranged from 1.56 ppb to 15.88 ppb (figure 4(e)). Results indicate a positive correlation between exhaled breath and indoor air VC concentrations (Pearson correlation coefficient = 0.8891). Linear regression analysis yielded a relationship between exhaled breath and indoor air concentrations (figure 4(f), $y=0.1233x,R^{2}=0.9005$).

## 4. Discussion

This study evaluated exposure to PCE and its degradation products in a community setting by measuring exhaled breath and indoor air using the PTR-TOF-MS, which is a highly sensitive, real-time instrument. The PTR-TOF-MS can detect VOCs at low concentrations, which is beneficial for environmental studies where levels may be below the detection limits of other instruments. Its portability allows for on-site sample analysis, reducing potential sample loss that could occur with conventional laboratory methods in which samples need to be transported to a laboratory. In this study, exhaled breath samples were collected from 73 participants on weekend mornings and analyzed within several hours of sample collection. PCE was detected in 66 samples, TCE in 26 samples, and VC in 68 samples. Average concentrations were compared based on participants’ residence locations, categorized as within the Superfund Site, Plume A, Plume B, Plume C, or Outside the Plume areas. Surprisingly, average concentrations of PCE, TCE, and VC were highest in the Outside the Plume areas. To monitor diurnal changes, exhaled breath samples from 6 participants were measured 3–4 times during the daytime. Temporal variations in these samples were statistically insignificant, indicating no significant changes in exhaled breath concentrations throughout the day. Correlations between exhaled breath and indoor air were evaluated by measuring 13 participants and their bedrooms. PCE, TCE, and VC concentrations in exhaled breath showed positive correlations with those in indoor air.

Notable concentrations detected in the Outside the Plume areas suggest multiple pathways of exposure, indicating that individuals might be influenced by various environments that they inhabit during the daytime [66]. The variability in soil-vapor intrusion and building ventilation conditions across participant homes may also contribute to differences in indoor air concentrations. Older homes may have lower mechanical ventilation capacity and more compromised foundations compared to newer homes which could contribute to higher indoor air concentrations. In future work, our research group is going to investigate the historical variations in PCE concentrations found in the groundwater, indoor air, and soil samples across the four plume areas using data collected from IDEM, U.S. EPA, and other environmental consulting firms.

PCE degradability also needs to be considered in community-based PCE exposure investigations. The concentration range of PCE in exhaled breath measured in this study (mean: 0.26 ppb (1.78 *μ*g m^−3^), range: 0.01 ppb (0.07 *μ*g m^−3^)—3.75 ppb (25.44 *μ*g m^−3^)) and PCE in indoor air measured (mean: 0.16 ppb (1.05 *μ*g m^−3^), range: 0.09 ppb (0.61 *μ*g m^−3^)—0.23 ppb (1.56 *μ*g m^−3^)) were low compared to previously reported results. Liu *et al* reported in our previous study PCE was detected in 39 exhaled breath samples (mean: 6.6 *μ*g m^−3^; range: 1.9–44 *μ*g m^−3^) and a total of six out of nine homes were detected with PCE concentrations ranging from 1.6 to 70 g m^−3^ [17]. In addition, a comparison of the Superfund Site with other plumes shows that the Outside the Plume had the highest average PCE concentration in the exhaled breath of residents. This shows the extent of current exposure, and because PCE is biodegradable and can degrade under certain conditions, the degradation of PCE over time is an important aspect to consider when interpreting results. Scheepers *et al* detected both PCE and its degradation products in exhaled breath from workers exposed to PCE [35]. In addition, co-exposure to PCE and TCE have been observed in previous studies [67]. This investigation demonstrates that co-exposure to PCE and VC was common among the residents of Martinsville, and to a lesser extent, PCE and TCE co-exposure. PCE, TCE and VC are neurotoxic chemicals [68–71], and potential adverse neurotoxic synergistic effects have also been reported [67].

To overcome resource limitations and obtain comprehensive data, future studies should develop criteria for selecting participants, such as proximity to hazardous waste sites, length of residency, and age. Since participation was voluntary, self-selection bias may arise, as individuals who suspect contamination could be more inclined to volunteer for testing [19]. In addition, based on observations from our community partners, parents who were more concerned about PCE contamination in the community were more likely to let their children to participate in the study, compared with those who might be less aware about the contamination. This created an education inequity which potentially impacted recruitment and sample distribution. Increasing the sample size and randomizing participants will enhance the robustness of conclusions. The sample size limitation in plume regions may impact the generalizability of these findings. Future studies with larger sample size and more diverse participants can enhance the robustness of the conclusions drawn from this research. In addition, the inclusion of participants from various demographics and geographical locations would contribute to a more comprehensive understanding of exposure patterns for PCE and its degradation products.

This study confirmed the feasibility of using PTR-TOF-MS for measuring PCE concentrations in exhaled breath and indoor air in community-based investigations. Future research should focus on historical variations in PCE concentrations, additional exposure sources, and the impact of PCE exposure on neurological health outcomes. The positive correlation between indoor air and exhaled breath PCE concentrations, as well as the similarity in patterns among siblings, indicates significant exposure from the external environment. Other potential sources, such as non-dry cleaning industries and household consumer products could influence individual exposure levels. PCE has been utilized in various fields, including: (1) feedstock for manufacturing chlorinated chemicals, (2) degreasers and solvent cleaners, (3) dry cleaning and textile processing, (4) carpets and spot cleaning, (5) industrial catalyst regeneration, (6) lubricants, (7) wood furniture manufacturing, (8) sealants and adhesives, (9) metal and stone polishes and coatings, (10) paints, inks, and ink removal products, (11) metal surface preparation and cleaning, (12) plastic and rubber manufacturing, (13) laboratory applications, (14) automotive manufacturing and maintenance, (15) mold cleaners, releases, and protectants, and (16) various other industrial, commercial, and consumer uses [72]. Future research should explore these sources comprehensively to provide a more nuanced understanding of PCE exposure pathways.

## 5. Conclusion

This study examined PCE, TCE, and VC exposures in a community setting by analyzing exhaled breath and indoor air using a PTR-TOF-MS. By focusing on Martinsville, Indiana, a location with multiple PCE contamination sites, including a designated Superfund site, the study showed a positive correlation between PCE, TCE, and VC concentrations in exhaled breath and those chemicals in indoor air. The findings of this study demonstrate the feasibility of using exhaled breath analysis to evaluate PCE exposures in a community setting. Overcoming the challenges of field studies, addressing sample size limitations, understanding PCE degradation, and exploring other potential sources are crucial steps toward developing effective strategies to mitigate PCE exposures and safeguard public health.

## Figures and Tables

**Table 1.** PCE, TCE, VC concentrations in exhaled breath during the daytime for six participants.

Participant ID	1st Sample (ppb)	2nd Sample (ppb)	3rd Sample (ppb)	4th Sample (ppb)	Average (ppb)	SD* (ppb)
PCE						
MV018	3.20	2.72	2.33	2.90	2.79	0.36
MV031	0.13	0.15	0.16	0.16	0.15	0.02
MV032	0.13	0.13	0.16	0.15	0.14	0.01
MV037	<MDL**	0.13	0.16	0.15	0.14	0.01
MV057	0.15	0.15	0.15	-***	0.15	0.00
MV058	0.15	0.16	0.16	—	0.16	0.01
TCE						
MV018	0.15	0.16	0.14	0.18	0.15	0.02
MV031	<MDL	0.13	0.18	0.14	0.15	0.03
MV032	<MDL	0.16	<MDL	0.15	0.15	0.01
MV037	0.14	<MDL	<MDL	0.11	0.13	0.02
MV057	0.28	0.20	0.22	NA	0.23	0.04
MV058	0.19	0.17	0.17	NA	0.18	0.01
VC						
MV018	1.23	0.77	1.35	1.30	1.16	0.27
MV031	3.09	1.90	1.84	2.18	2.25	0.58
MV032	1.44	2.08	1.67	2.06	1.81	0.31
MV037	1.95	3.09	1.73	1.60	2.09	0.68
MV057	4.95	4.31	5.07	—	4.78	0.41
MV058	1.86	2.09	1.72	—	1.89	0.19

SD*: Standard Deviation.

MDL**: Method Detection Limit (0.11 ppb).

-***: Not collected.

## Data Availability

The data that support the findings of this study are available upon reasonable request from the authors.
